# Do Viruses Exchange Genes across Superkingdoms of Life?

**DOI:** 10.3389/fmicb.2017.02110

**Published:** 2017-10-31

**Authors:** Shahana S. Malik, Syeda Azem-e-Zahra, Kyung Mo Kim, Gustavo Caetano-Anollés, Arshan Nasir

**Affiliations:** ^1^Department of Biosciences, COMSATS Institute of Information Technology, Islamabad, Pakistan; ^2^Division of Polar Life Sciences, Korea Polar Research Institute, Incheon, South Korea; ^3^Evolutionary Bioinformatics Laboratory, Department of Crop Sciences, University of Illinois at Urbana-Champaign, Urbana, IL, United States

**Keywords:** virus host, protein structure, fold superfamily, comparative genomics, horizontal gene transfer, virus evolution

## Abstract

Viruses can be classified into archaeoviruses, bacterioviruses, and eukaryoviruses according to the taxonomy of the infected host. The host-constrained perception of viruses implies preference of genetic exchange between viruses and cellular organisms of their host superkingdoms and viral origins from host cells either via escape or reduction. However, viruses frequently establish non-lytic interactions with organisms and endogenize into the genomes of bacterial endosymbionts that reside in eukaryotic cells. Such interactions create opportunities for genetic exchange between viruses and organisms of non-host superkingdoms. Here, we take an atypical approach to revisit virus-cell interactions by first identifying protein fold structures in the proteomes of archaeoviruses, bacterioviruses, and eukaryoviruses and second by tracing their spread in the proteomes of superkingdoms Archaea, Bacteria, and Eukarya. The exercise quantified protein structural homologies between viruses and organisms of their host and non-host superkingdoms and revealed likely candidates for virus-to-cell and cell-to-virus gene transfers. Unexpected lifestyle-driven genetic affiliations between bacterioviruses and Eukarya and eukaryoviruses and Bacteria were also predicted in addition to a large cohort of protein folds that were universally shared by viral and cellular proteomes and virus-specific protein folds not detected in cellular proteomes. These protein folds provide unique insights into viral origins and evolution that are generally difficult to recover with traditional sequence alignment-dependent evolutionary analyses owing to the fast mutation rates of viral gene sequences.

## Introduction

Depending on the nature of the infected host, viruses can be broadly classified into three major groups, *archaeoviruses, bacterioviruses* (Krupovic et al., 2016), and *eukaryoviruses*, in addition to the lesser-known virophages that parasitize giant viruses (La Scola et al., 2003, 2008). While host jumps are common (Longdon et al., 2014; Geoghegan et al., 2017), such as HIV from chimps (Sharp and Hahn, 2010), SARS Coronavirus from bats (Li et al., 2005), H1N1 from birds (Webby and Webster, 2001), and arboviruses that replicate in mammalian cells and insect vectors, viruses are not known to infect cellular organisms separated by superkingdom (domain of life) boundaries (Nasir et al., 2014, 2017). This has been confirmed by recent studies revealing strong biases in the distribution of viral replicon types in superkingdoms such as the paucity of discovered RNA and retrotranscribing viruses in prokaryotes and their abundance and diversity in eukaryotic species such as mammals and vertebrates (Nasir et al., 2014; Koonin et al., 2015). The highly specific nature of virus-host interactions logically constrains genetic exchange to occur more frequently between the interacting partners. For example, *bacterioviruses* are known to capture bacterial genes involved in toxins and photosynthesis (Canchaya et al., 2004; Lindell et al., 2004). Similarly, *eukaryoviruses* often capture genes involved in antiviral immunity from eukaryotic cells (Elde and Malik, 2009; Rappoport and Linial, 2012). Thus, host-constrained evolution of viral lineages has led to favoring either the “escape” or “reduction” models for the origin of modern viruses, both attributing viral origins from modern or ancient host cells (reviewed in Hendrix et al., 2000; Forterre and Krupovic, 2012; Nasir et al., 2012b).

Virus-host affiliations however are largely established by observing the cytopathic effects of viral infection or by microscopy detection of virion particles. These properties relate to the lytic mode of viral reproduction that has historically remained on focus due to the noxious effects that lysis has on human health, livestock, and agriculture. However, viruses can also frequently endogenize by integration into cellular genomes (Feschotte and Gilbert, 2012), sometimes providing useful novel genes to make them evolutionarily competitive (Cornelis et al., 2012). Moreover, many viruses either infect bacterial symbionts of eukaryotic cells (e.g., the bacterial component of the human microbiota, Turnbaugh et al., 2007) or reside as prophages in the genomes of obligate intracellular bacteria that infect a wide range of eukaryotic hosts (Brüssow et al., 2004). These virus-cell interactions are largely non-lytic in nature and because they do not yield the classic phenotypic effects of viral infection, have likely remained underestimated through established methods of virus discovery (reviewed in Nasir et al., 2017). Importantly, such interactions blur the traditional concept of “virus host” and raise the possibility of viruses interacting (not necessarily in a lytic manner) and exchanging genetic material simultaneously with more than one superkingdom of life. Bordenstein and Bordenstein (2016) recently reported an example of a eukaryotic gene module in bacteriophage WO residing as prophage in the intracellular α-proteobacterium *Wolbachia*, which infects a large group of insects. In order to produce viral progeny, the bacteriophage WO must neutralize antiviral defense and enter/exit the membranes of both bacterial and eukaryal organisms (Bordenstein and Bordenstein, 2016). The study therefore offered unique insights into virus-cell interactions that extend beyond their known hosts and identified viruses of endosymbiotic bacteria as interesting examples of vectors with genetic material from non-host superkingdoms.

Here we take a comparative genomic approach to revisit virus-cell interactions by identifying the repertoires of protein structural domains (proteomes) in 3,440 viruses categorized into *archaeoviruses, bacterioviruses*, and *eukaryoviruses* and tracing their spread in the proteomes of 1,620 “hosts” from Archaea, Bacteria, and Eukarya. Protein domains were grouped into fold superfamilies (FSFs), as defined by the structural classification of proteins (SCOP) database (Andreeva et al., 2008; Fox et al., 2014) to include distantly related domains that show negligible sequence identity (can be < 15%) but recognizable common three-dimensional (3D) cores and biochemical functions that are likely indicative of shared ancestry. The evolutionary conservation of FSFs makes them useful molecular characters for inferring long-term viral evolutionary patterns, especially since fast mutational rates of viral gene sequences (Sanjuán et al., 2010) sometimes prohibit meaningful global evolutionary analyses (Abroi and Gough, 2011; Caetano-Anollés and Nasir, 2012; Nasir and Caetano-Anollés, 2015).

**Figure 1:**
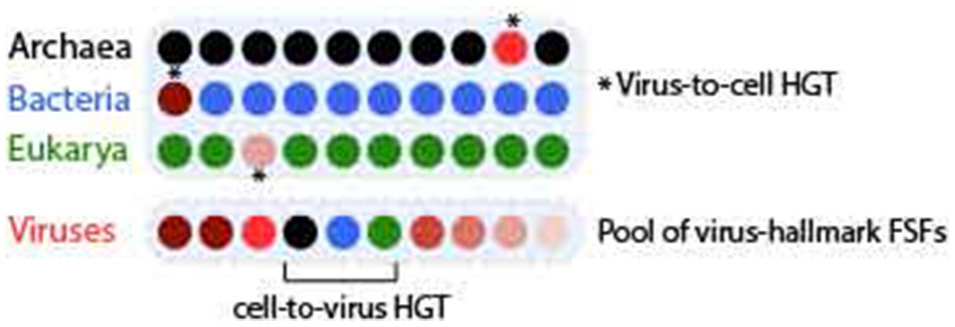
Demonstration of virus-to-cell and cell-to-virus HGT events. Ten genomes are displayed as colored closed disks each for Archaea (black), Bacteria (blue), Eukarya (green), and viruses. Seven out of 10 viral genomes encode different virus hallmark FSFs (with incidence represented by different shades of red) such as those involved in virion synthesis and capsid assembly. If any of these virus-hallmark FSFs is detected in no more than 1/10 cellular genomes (real *f*-values are even lower), the event is determined to be virus-to-cell HGT. In turn, any of the cellular FSFs that are widespread in cells (i.e., present in 9/10 cellular genomes) are detected in a viral genome, that event is determined to be cell-to-virus HGT.

The comparative exercise of tracing the spread of each viral FSF in cellular proteomes was made explicit with an *f*-value representing the fraction of cellular proteomes encoding individual FSFs (see Methods). The *f*-values of viral FSFs in cellular proteomes and their reported biochemical functions were then used to postulate hypotheses regarding the direction of gene transfer, virus-to-cell or cell-to-virus (see Figure 1 for demonstration). For example, an FSF with a viral hallmark function (e.g., virion synthesis) that had negligible presence in proteomes of a cellular superkingdom (e.g., *f* < 1%) was considered a candidate for horizontal gene transfer (HGT) event from virus-to-cell rather than from cell-to-virus as the latter would require invoking multiple gene loss events in related cellular species. This approach of inferring the likely direction of gene transfer is thus similar to considering anomalous phylogenetic distributions of genes in closely related species as more likely a result of HGT rather than vertical inheritance and loss. This method reliably detects HGT events (Philippe and Douady, 2003), especially in viral genes where sequence identity with cellular counterparts may be too low to produce meaningful alignment-dependent phylogenetic trees (Nasir and Caetano-Anollés, 2015). The tracings yielded unique insights into genetic transfers between viruses and cells, highlighted the quantitatively greater cross-superkingdom genetic exchange occurring between *bacterioviruses* and eukaryotes and *eukaryoviruses* and bacteria, and supported models of viral origins from ancient cells (Nasir et al., 2012b). The genetic crosstalk between viral and cellular proteomes that we uncover with this comparative genomics approach presents a more global picture for evolutionary understanding of virus-cell interactions that goes beyond the perceived textbook definitions of virus hosts (Nasir et al., 2017).

## Results

### A large number of protein folds shared exclusively between viruses and their host genomes were likely transferred from viruses to cells

**Figure 2:**
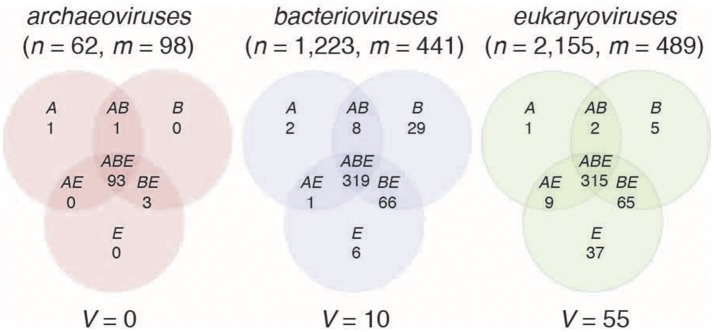
Sharing of protein structural domains between viral and cellular proteomes. The Venn diagrams illustrate the number of FSFs detected in the proteomes of *archaeoviruses, bacterioviruses*, and *eukaryoviruses* and their distributions in the proteomes of superkingdoms Archaea (A), Bacteria (B), and Eukarya (E). *n* = total number of viral proteomes, *m* = total number of FSFs detected in viral proteomes. V represents virus-specific FSFs (Table 2).

A total of 98, 441, and 489 FSFs were detected in the proteomes of 62 *archaeoviruses*, 1,223 *bacterioviruses*, and 2,155 *eukaryoviruses* (Table S1), respectively (Figure 2). Based on the presence/absence of these viral FSFs in 1,620 cellular proteomes from Archaea (122 in number), Bacteria (1,115), and Eukarya (383), seven mutually exclusive Venn groups could be defined each for *archaeoviruses, bacterioviruses*, and *eukaryoviruses*: *A* (viral FSFs shared only with archaeal proteomes), *B* (shared only with bacterial proteomes), *E* (shared only with eukaryotic proteomes), *AB* (shared only with prokaryotic proteomes), *AE* (shared only with archaeal and eukaryal proteomes), *BE* (shared only with bacterial and eukaryal proteomes), and *ABE* (shared with proteomes of all three superkingdoms), in addition to virus-specific (*V*) FSFs not detected in cellular proteomes (Figure 2).

Under the expected host-constrained evolution model of viral lineages, viruses of any superkingdom are expected to share more FSFs with organisms of their host superkingdom rather than with organisms of other superkingdoms. Indeed, *archaeoviruses* shared a single FSF exclusively with Archaea (Venn group *A*; Hypothetical protein D-63) but none exclusively with Bacteria (group *B*) and Eukarya (group *E*) (Figure 2, Table 1). In turn, *bacterioviruses* shared 29 FSFs exclusively with Bacteria (group *B*) but also 2 and 6 FSFs with Archaea and Eukarya in groups *A* and *E*, respectively. Similarly, *eukaryoviruses* shared 37 FSFs exclusively with Eukarya (group *E*) but also 1 and 5 FSFs with Archaea and Bacteria in groups *A* and *B*, respectively (Figure 2, Table 1). At first glance, the data support the idea that viruses tend to share/exchange more genes with their host groups relative to organisms they do not infect or associate with.

**Table 1:** Virus-Host FSF sharing.

**SCOP Id**	**SCOP *ccs***	**FSF description**	***f*-value (A)**	***f*-value (B)**	***f*-value (E)**	***f*-value (AV)**	***f*-value (BV)**	***f*-value (EV)**
**FSFS ONLY IN *ARCHAEOVIRUSES* AND ARCHAEAL PROTEOMES (*n* = 1)**								
109801	a.30.5	Hypothetical protein D-63	0.0082	0.0000	0.0000	0.0968	0.0000	0.0000
**FSFS ONLY IN *BACTERIOVIRUSES* AND BACTERIAL PROTEOMES (*n* = 29)**								
160570	d.368.1	YonK-like	0.0000	0.0018	0.0000	0.0000	0.0008	0.0000
64210	d.186.1	Head-to-tail joining protein W, gpW	0.0000	0.0170	0.0000	0.0000	0.0123	0.0000
159865	d.186.2	XkdW-like	0.0000	0.0054	0.0000	0.0000	0.0049	0.0000
54857	d.57.1	DNA damage-inducible protein DinI	0.0000	0.0520	0.0000	0.0000	0.0090	0.0000
51327	b.90.1	Head-binding domain of phage P22 tailspike protein	0.0000	0.0135	0.0000	0.0000	0.0074	0.0000
143749	d.323.1	Phage tail protein-like	0.0000	0.0278	0.0000	0.0000	0.0098	0.0000
89064	a.179.1	Replisome organizer (g39p helicase loader/inhibitor protein)	0.0000	0.0009	0.0000	0.0000	0.0016	0.0000
54328	d.15.5	Staphylokinase/streptokinase	0.0000	0.0036	0.0000	0.0000	0.0041	0.0000
56826	e.27.1	Upper collar protein gp10 (connector protein)	0.0000	0.0009	0.0000	0.0000	0.0147	0.0000
46575	a.237.1	DNA polymerase III theta subunit-like	0.0000	0.0493	0.0000	0.0000	0.0025	0.0000
140919	a.263.1	DNA terminal protein	0.0000	0.0009	0.0000	0.0000	0.0025	0.0000
159871	d.230.6	YdgH-like	0.0000	0.0502	0.0000	0.0000	0.0016	0.0000
68918	a.140.4	Recombination endonuclease VII, C-terminal and dimerization domains	0.0000	0.0009	0.0000	0.0000	0.0311	0.0000
160582	d.100.2	MbtH-like	0.0000	0.1623	0.0000	0.0000	0.0008	0.0000
141658	b.163.1	Bacteriophage trimeric proteins domain	0.0000	0.0027	0.0000	0.0000	0.0139	0.0000
51274	b.85.2	Head decoration protein D (gpD, major capsid protein D)	0.0000	0.0072	0.0000	0.0000	0.0090	0.0000
58046	h.1.17	Fibritin	0.0000	0.0009	0.0000	0.0000	0.0417	0.0000
58059	h.2.1	Tetramerization domain of the Mnt repressor	0.0000	0.0027	0.0000	0.0000	0.0041	0.0000
**50789**	**b.57.1**	**Herpes virus serine proteinase, assemblin**	**0.0000**	**0.0682**	**0.0000**	**0.0000**	**0.0147**	**0.0255**
50017	b.32.1	gp9	0.0000	0.0009	0.0000	0.0000	0.0581	0.0000
58091	h.4.2	Clostridium neurotoxins, “coiled-coil” domain	0.0000	0.0018	0.0000	0.0000	0.0008	0.0000
57987	h.1.4	Inovirus (filamentous phage) major coat protein	0.0000	0.0099	0.0000	0.0000	0.0074	0.0000
101059	a.159.3	B-form DNA mimic Ocr	0.0000	0.0009	0.0000	0.0000	0.0123	0.0000
158668	a.285.1	MtlR-like	0.0000	0.0753	0.0000	0.0000	0.0008	0.0000
103370	d.262.1	NinB	0.0000	0.0386	0.0000	0.0000	0.0368	0.0000
118010	d.64.2	TM1457-like	0.0000	0.2161	0.0000	0.0000	0.0057	0.0000
48657	a.136.1	FinO-like	0.0000	0.1686	0.0000	0.0000	0.0033	0.0000
50610	b.48.1	mu transposase, C-terminal domain	0.0000	0.0700	0.0000	0.0000	0.0139	0.0000
47681	a.49.1	C-terminal domain of B transposition protein	0.0000	0.0135	0.0000	0.0000	0.0025	0.0000
**FSFS ONLY IN *EUKARYOVIRUSES* AND EUKARYAL PROTEOMES (*n* = 55)**								
58069	h.3.2	Virus ectodomain	0.0000	0.0000	0.0757	0.0000	0.0000	0.0362
90229	g.66.1	CCCH zinc finger	0.0000	0.0000	1.0000	0.0000	0.0000	0.0074
**49749**	**b.121.2**	**Group II dsDNA viruses VP**	**0.0000**	**0.0000**	**0.0131**	**0.0000**	**0.0008**	**0.0381**
101912	b.69.12	Sema domain	0.0000	0.0000	0.3211	0.0000	0.0000	0.0060
57567	g.22.1	Serine protease inhibitors	0.0000	0.0000	0.3316	0.0000	0.0000	0.0023
54117	d.9.1	Interleukin 8-like chemokines	0.0000	0.0000	0.1540	0.0000	0.0000	0.0084
47836	a.61.1	Retroviral matrix proteins	0.0000	0.0000	0.0366	0.0000	0.0000	0.0186
50353	b.42.1	Cytokine	0.0000	0.0000	0.3264	0.0000	0.0000	0.0292
52087	c.13.1	CRAL/TRIO domain	0.0000	0.0000	0.9948	0.0000	0.0000	0.0005
103417	e.48.1	Major capsid protein VP5	0.0000	0.0000	0.0026	0.0000	0.0000	0.0241
56994	g.1.1	Insulin-like	0.0000	0.0000	0.3055	0.0000	0.0000	0.0009
57535	g.18.1	Complement control module/SCR domain	0.0000	0.0000	0.3760	0.0000	0.0000	0.0125
57180	g.3.8	Cellulose-binding domain	0.0000	0.0000	0.2846	0.0000	0.0000	0.0005
161008	e.76.1	Viral glycoprotein ectodomain-like	0.0000	0.0000	0.0131	0.0000	0.0000	0.0390
54277	d.15.2	CAD & PB1 domains	0.0000	0.0000	0.9530	0.0000	0.0000	0.0023
47195	a.24.5	TMV-like viral coat proteins	0.0000	0.0000	0.0418	0.0000	0.0000	0.0190
82856	e.42.1	L-A virus major coat protein	0.0000	0.0000	0.0104	0.0000	0.0000	0.0019
158235	a.271.1	SOCS box-like	0.0000	0.0000	0.3159	0.0000	0.0000	0.0005
47943	a.73.1	Retrovirus capsid protein, N-terminal core domain	0.0000	0.0000	0.0522	0.0000	0.0000	0.0190
47353	a.28.3	Retrovirus capsid dimerization domain-like	0.0000	0.0000	0.1723	0.0000	0.0000	0.0125
**88645**	**b.121.5**	**ssDNA viruses**	**0.0000**	**0.0000**	**0.0418**	**0.0000**	**0.0139**	**0.0320**
101399	a.206.1	P40 nucleoprotein	0.0000	0.0000	0.0104	0.0000	0.0000	0.0005
110132	b.147.1	BTV NS2-like ssRNA-binding domain	0.0000	0.0000	0.0026	0.0000	0.0000	0.0046
49599	b.8.1	TRAF domain-like	0.0000	0.0000	0.9974	0.0000	0.0000	0.0005
57302	g.7.1	Snake toxin-like	0.0000	0.0000	0.3211	0.0000	0.0000	0.0005
50122	b.34.7	DNA-binding domain of retroviral integrase	0.0000	0.0000	0.0235	0.0000	0.0000	0.0097
140809	a.260.1	Rhabdovirus nucleoprotein-like	0.0000	0.0000	0.0183	0.0000	0.0000	0.0125
46919	a.4.10	N-terminal Zn binding domain of HIV integrase	0.0000	0.0000	0.0261	0.0000	0.0000	0.0084
57924	g.52.1	Inhibitor of apoptosis (IAP) repeat	0.0000	0.0000	0.7441	0.0000	0.0000	0.0376
57933	g.53.1	TAZ domain	0.0000	0.0000	0.4700	0.0000	0.0000	0.0005
103575	g.16.2	Plexin repeat	0.0000	0.0000	0.3316	0.0000	0.0000	0.0023
57059	g.3.6	omega toxin-like	0.0000	0.0000	0.0444	0.0000	0.0000	0.0014
140586	a.242.1	Dcp2 domain-like	0.0000	0.0000	0.9034	0.0000	0.0000	0.0005
57501	g.17.1	Cystine-knot cytokines	0.0000	0.0000	0.3185	0.0000	0.0000	0.0046
69340	b.80.5	C-terminal domain of adenylylcyclase associated protein	0.0000	0.0000	0.9765	0.0000	0.0000	0.0009
49830	b.20.1	ENV polyprotein, receptor-binding domain	0.0000	0.0000	0.0313	0.0000	0.0000	0.0042
81382	a.157.1	Skp1 dimerisation domain-like	0.0000	0.0000	0.9687	0.0000	0.0000	0.0032

*FSFs shared exclusively between the proteomes of host superkingdoms, Archaea (A), Bacteria (B), and Eukarya (E), and the proteomes of their viruses, archaeoviruses (AV), bacterioviruses (BV), and eukaryoviruses (EV). FSFs are identified both by SCOP numeric IDs and alpha-numeric concise classification strings (ccs). FSF distribution (f-values, number of proteomes in a superkingdom or virus group encoding an FSF/total number of proteomes in that superkingdom or virus group) are also listed. FSF b.57.1 was also detected in eukaryoviruses in addition to Bacteria and bacterioviruses and FSFs b.121.2 and b.121.5 were also detected in bacterioviruses in addition Eukarya and eukaryoviruses possibly indicating genetic crosstalk or ancient ancestry (read text). These FSFs are highlighted in bold*.

Remarkably, the 29 FSFs shared exclusively between bacterioviral and bacterial proteomes included several viral hallmark proteins involved in phage (virus) assembly such as the gp9 and gp10 proteins, head-binding, head-to-tail joining, head decoration, and tail proteins, along with the major coat proteins of ssDNA harboring bacterioviruses (*Inoviridae*) and the dimerization domain of bacteriophage T4 recombination endonuclease VII (Table 1). In addition, the coiled-coiled domain of bacterial neurotoxin involved in host virulence was also detected. Interestingly, the majority of FSFs in group *B* had *f*-values close to 0 indicating their rare presence in bacterial proteomes (Table 1). Collectively, therefore, the enrichment of the *B* Venn group in viral hallmark functions with negligible presence in bacterial proteomes suggests that these genes were likely acquired by bacterial cells from viruses via virus-to-cell HGT, a phenomenon that has been assumed to be relatively less frequent than cell-to-virus HGT (Moreira and Lopez-Garcia, 2009), though now increasingly being revisited (Forterre, 2016). Similarly, viral hallmark proteins such as the viral capsid and coat-related proteins (e.g., the “jelly-roll” and “double jelly-roll” folds) (Abrescia et al., 2012), viral glycoproteins and matrix proteins, the integrase proteins of retroviruses and HIV, and toxins were part of the 37 FSFs shared exclusively between *eukaryoviruses* and Eukarya (the *E* Venn group) with low *f*-values in eukaryal proteomes (Table 1). These viral hallmark proteins shared exclusively between *eukaryoviruses* and eukaryotes could therefore also represent episodes of virus-to-cell gene transfer. In turn, other *E* FSFs such as the CCCH zinc finger domains (involved in regulation and DNA binding), CAD and PB1 domains (cell cycle and apoptosis), CRAL/TRIO domains (likely functional components of the visual cycle), TRAF-domain like (involved in stress response, immunity, apoptosis, among other roles), and others were near ubiquitous in eukaryotic proteomes (i.e., *f*-value close to 1.0, Table 1). These “cell-like” proteins detected in *eukaryoviruses* could therefore suggest recent gene capture by viruses from cells (i.e., cell-to-virus HGT) as likely part of viral mimicry of cellular proteins to interfere with the antiviral response (Elde and Malik, 2009).

In summary, a large number of FSFs shared exclusively between viruses and their host genomes had rare presence in hosts and were involved in virus-hallmark functions suggesting these genes likely originated in viral lineages and were later transferred to their host cells.

### Traces of genetic crosstalk between viruses and non-host superkingdoms could be recovered from the comparative genomic data

While the data of Figure 2 indicated significant levels of genetic exchange restricted between viruses and their known host superkingdoms, some bacterioviral and eukaryoviral FSFs were also shared with Eukarya and Bacteria, respectively (*archaeoviruses* shared no domains exclusively with either Bacteria or Eukarya) (Figure 2, Tables S2–S4). For example, *bacterioviruses* shared 2 FSFs exclusively with Archaea (group *A*) and 6 with Eukarya (group *E*) (Table S3). Interestingly, 4/6 *E* FSFs in *bacterioviruses* could be considered viral hallmark proteins such as FSFs b.121.2 (the “double jelly-roll” fold hallmark of capsid proteins of the PRD1/Adenovirus-like lineage) (Bamford, 2003; Abrescia et al., 2012), b.121.5 (the “jelly-roll” fold in ssDNA viruses members of the Picornavirus-like lineage), d.85.1 (capsid/coat related fold in RNA bacteriophages), and a.251.1 (the phage replication organizer domain) (Table S3). Viruses have been recently (re)-classified into structure-based lineages based on 3D structural similarities in capsid/coat architectures or common principles of functional virion construction (Bamford, 2003; Abrescia et al., 2012; Nasir and Caetano-Anollés, 2017). Some of the lineages such as the PRD1/Adenovirus-like lineage (characterized by the so-called “double jelly-roll” fold) include member viruses infecting the three cellular superkingdoms (Bamford, 2003; Abrescia et al., 2012). Thus, it is no surprise that *bacterioviruses* share capsid/coat related protein folds characteristic of *eukaryoviruses*. It is however indeed intriguing to note that these FSFs were present in eukaryotic proteomes, especially because the capsid is considered to be a virus hallmark (Benson et al., 2004; Abrescia et al., 2010). Thus, rare occurrences of capsid/coat related genes in cellular proteomes are more likely due to virus-to-cell HGT or their utilization in the assembly of capsid-like architectures in cells (e.g., carboxysomes and protein microcompartments in prokaryotes, Yeates et al., 2007, 2011) that are hitherto believed to be rare in cells (Cheng and Brooks, 2013; Nasir and Caetano-Anollés, 2017).

In turn, *eukaryoviruses* shared a single FSF exclusively with Archaea (group *A*; Chromosomal protein MC1) and 5 FSFs exclusively with Bacteria (group *B*) (Table S4). The MC1 protein is associated with thermophilic archaeal species (*f*-value = 0.26 in Archaea) and is involved in protecting DNA denaturation at high temperature (Chartier et al., 1989). Its presence in *eukaryoviruses* (but not in eukaryotic proteomes!) is therefore intriguing and could signal undiscovered viral-mediated interactions between eukaryotic and archaeal species. In turn, the 5 FSFs shared exclusively between *eukaryoviruses* and bacterial proteomes (the *B* Venn group) included capsid proteins (Outer capsid protein sigma 3) and other virus and cell-like proteins likely indicating a mixed ancestry (Table S4).

Finally, the *BE* Venn group for *archaeoviruses*, the *AE* group for *bacterioviruses*, and the *AB* group for *eukaryoviruses* may also represent genetic exchanges occurring between viruses and non-host superkingdoms. For *archaeoviruses*, the d.285.1 (DNA-binding domain of intron-encoded endonucleases), a.118.25 (TROVE domain-like), and b.22.1 (TNF-like) FSFs were detected in the *BE* group. In turn, only one bacterioviral FSF (d.282.1, SSo0622-like) was detected in the *AE* group and 2 eukaryoviral FSFs were detected in the *AB* group (a.18.1, T4 endonuclease V and g.90.1, E6 C-terminal domain-like) (highlighted in Tables S2–S4). These FSFs are likely candidates of genetic transfer occurring between viruses and non-host superkingdoms, more likely in the cell-to-virus direction because of the “cell-like” nature of these FSFs.

### An unanticipated relatively greater genetic affiliation between *Bacterioviruses* and eukaryal proteomes and *Eukaryoviruses* and bacterial proteomes

**Figure 3:**
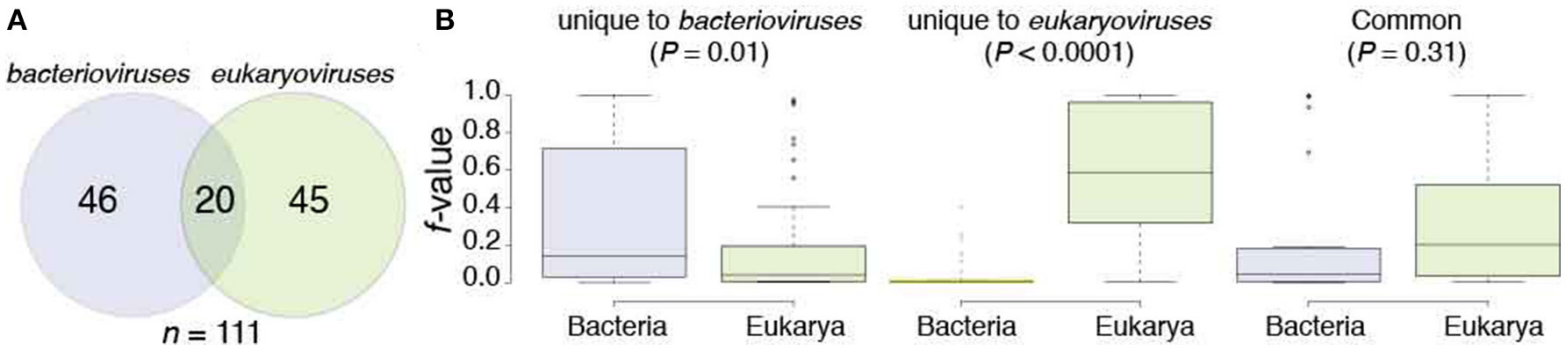
The structural domain composition of the *BE* Venn group in *bacterioviruses* and *eukaryoviruses*. **(A)** The Venn diagram describes how the *BE* FSFs were shared between *bacterioviruses* and *eukaryoviruses*. **(B)** Boxplots display the distribution of *f*-values (number of proteomes in a superkingdom encoding an FSF divided by the total number of proteomes in that superkingdom) for *BE* FSFs unique to *bacterioviruses*, unique to *eukaryoviruses*, and common to both the bacterial and eukaryal proteomes (see also Table S5). *P*-values were calculated from two-sample Welch *t*-tests.

*Bacterioviruses* shared 66 FSFs and *eukaryoviruses* shared 65 FSFs with both Bacteria and Eukarya (the *BE* groups), respectively, which constituted 15 and 13% of total bacterioviral and eukaryoviral FSFs, respectively (Figure 2). Only 20 *BE* FSFs overlapped and the remaining (46/66 in *bacterioviruses* and 45/65 in *eukaryoviruses*) were uniquely shared with bacterial and eukaryal proteomes (Figure 3A), thus extending the total number of *BE* FSFs to 111. The 46 *BE* FSFs unique to *bacterioviruses* (i.e., FSFs detected in bacterioviral, bacterial and eukaryal proteomes but not in eukaryoviral proteomes) were significantly more widespread in bacterial proteomes (Welch two-sample *t*-test, *P* = 0.01) while the 45 *BE* FSFs unique to *eukaryoviruses* (FSFs detected in eukaryoviral, bacterial and eukaryal proteomes but not in bacterioviral proteomes) were significantly more widespread in eukaryotic proteomes (*P* < 0.0001) with *f*-values approaching 1.0 in some cases (Figure 3B).

We hypothesize that *bacterioviruses* and *eukaryoviruses* acquired *BE* FSFs directly from their host cells (i.e., Bacteria and Eukarya, respectively) without the need to invoke genetic crosstalk between viruses and non-host superkingdoms and also between *bacterioviruses* and *eukaryoviruses* (since these FSFs were absent in one of the two viral groups except for the 20 common *BE* FSFs, Figure 3A). While, this may represent the generic trend, we noticed that 6 *BE* FSFs unique to *bacterioviruses* had an *f*-value that was 25% higher in eukaryotic proteomes than the corresponding *f*-value in bacterial proteomes (highlighted in Table S5). For example, FSF d.344.1 (PriA/YqbF domain) was present in roughly 95% of eukaryotic proteomes and in only 2% bacterial proteomes (i.e., *f*-value differential of 93%). For another 22 *BE* FSFs, the differential in *f*-values was either negligible or below the 25% cutoff making it difficult to establish the likely direction of origin (i.e., from Eukarya to *bacterioviruses* or from Bacteria to *bacterioviruses*). In fact, only 18 out of 46 *BE* FSFs in *bacterioviruses* had an *f*-value > 25% in bacterial proteomes than the corresponding *f*-value in eukaryotic proteomes (Table S5). Therefore, closer inspection of *BE* FSFs in *bacterioviruses* indicated that both sources of origin could be considered likely, especially when accounting for the relative preference of bacterial species to become endosymbionts of eukaryotes and considering mechanical similarities between bacterial and eukaryotic cells (read below). The same was also true for 20 *BE* FSFs common to *bacterioviruses* and *eukaryoviruses* where the *f*-value differential was under 25% for 11 out of 20 FSFs. However, 37 out of 45 *BE* FSFs in *eukaryoviruses* had an *f*-value of 25% or greater in eukaryotic proteomes than the corresponding *f*-value in bacterial proteomes (Table S5) indicating that *eukaryoviruses* perhaps did not engage in genetic exchange directly from Bacteria (or *bacterioviruses*).

**Figure 4:**
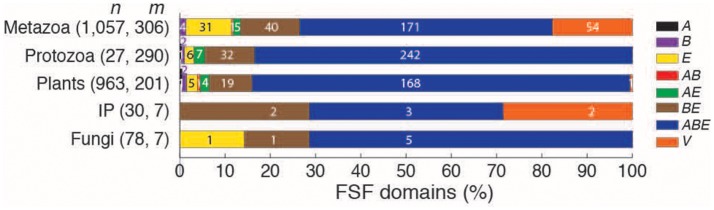
Breakdown of the 489 FSFs detected in *eukaryoviruses*. *Eukaryoviruses* were divided into viruses of plants (included all plants, blue-green algae, and diatoms), metazoa (vertebrates and invertebrates), protozoa (animal-like protists), fungi, and a group that includes invertebrates and plants (IP). For each subgroup, bars indicate the percentage of FSFs present in one of the seven Venn groups listed on the right (see also Figure 2) and the percentage of virus-specific FSFs. Numbers on bars indicate actual count. *n* = total number of viral proteomes, *m* = total number of FSFs detected in viral proteomes.

To test, we divided *eukaryoviruses* into five subgroups representing viruses of fungi, plants, metazoa, protozoa, and invertebrates-plants (viruses that can replicate in both plants and insect vectors), as defined by the NCBI Viral Genomes Resource (Figure 4). FSF distributions of the five subgroups of *eukaryoviruses* were mapped to the seven Venn groups already defined for *eukaryoviruses* (Figure 2). The majority of *eukaryoviruses* belonged to metazoa (*n* = 1,057) and plant hosts (963) revealing strong biases in the sequencing of human infection, livestock and agriculture related viruses. Interestingly, only 27 viruses were associated to protozoa. These viruses encoded a total of 291 FSFs (the second largest amongst the five eukaryoviral subgroups after 306 FSFs of metazoan viruses). This is expected since protozoa act as natural hosts of many “giant viruses” (e.g., *Acanthamoeba polyphaga*), which surpass parasitic cellular species both in particle and genome sizes and sometimes encode more than a thousand proteins (La Scola et al., 2003; Arslan et al., 2011; Philippe et al., 2013; Legendre et al., 2015). However, out of the total 65 *BE* FSFs detected in *eukaryoviruses* (Figure 2), 40 (62%) were detected in metazoan viruses and 32 (49%) in protozoan viruses (overlap of 14 common FSFs) (Figure 4). Animals are known hosts for symbiotic bacteria and also harbor large microbiota communities, especially in the gastrointestinal tract that is considered to be a “melting pot” for HGT (Shterzer and Mizrahi, 2015). Similarly, free-living amoeba (e.g., *Acanthamoeba*) are notorious reservoirs for both facultative and obligate intracellular bacteria and serve as “training grounds” to facilitate bacterial adaptation in eukaryotic cells (Barker and Brown, 1994; Molmeret et al., 2005). These two eukaryotic host subgroups therefore provide ample opportunities for *eukaryoviruses* to exchange genetic material either directly with bacterial proteomes or through prophages integrated in bacterial genomes.

### The ABE and virus-specific protein folds (V) provide unique insights into viral origins and evolution

**Figure 5:**
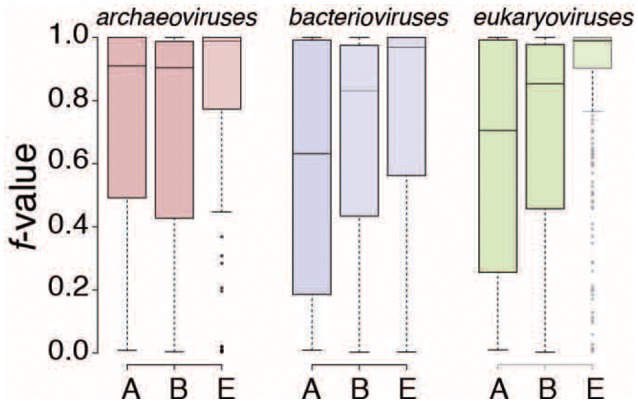
*ABE* FSFs are widespread in the proteomes of Archaea, Bacteria, and Eukarya. The *f*-value (number of proteomes in a superkingdom encoding an FSF / total number of proteomes in that superkingdom) distribution is plotted for the *ABE* Venn group of FSF domains for *archaeoviruses, bacterioviruses*, and *eukaryoviruses*. The three boxplots in each viral group describe FSF spread individually for Archaea (A), Bacteria (B), and Eukarya (E).

The *ABE* group was the largest Venn group for viruses of the three superkingdoms (i.e., 93 *ABE* FSFs out of 98 total FSFs in *archaeoviruses*, 319 out of 441 in *bacterioviruses*, and 315 out of 449 in *eukaryoviruses*, Figure 2). The *ABE* domains are, by definition, detected in the proteomes of all three superkingdoms and are more likely to evolve vertically and hold a deep history (Nasir and Caetano-Anollés, 2013, 2015). Indeed, *ABE* domains were widespread in cellular species (median *f*-value > 0.6 for all, Figure 5) and were enriched in “cell-like” functions such as metabolism, information, DNA repair, among others, and occasionally viral proteins (Tables S2–S4). Therefore, the *ABE* group stands in contrast to the “viral-like” nature of the other Venn groups, especially, *B* and *E* FSFs that had limited spread in cellular proteomes (Table 1). The presence of a large number of universal “cell-like” proteins in viral proteomes is therefore intriguing and worthy of exploration. It suggests two possible scenarios. First, the detection of *ABE* FSFs in viral proteomes effectively transforms *ABE* into an *ABEV* group, which now represents a large core of (near)-universal FSF domains shared by both cells and viruses. The mere existence of this FSF core supports an early “cell-like” phase in the evolution of modern viruses, an idea that has recently become popular (Nasir et al., 2012a,b) following the discovery of several “giant viruses” that overlap parasitic cells in physical and genome size (La Scola et al., 2003; Philippe et al., 2013; Legendre et al., 2014, 2015). Under this proposal, viruses are secondarily acellular as they either “escaped” or “reduced” from primordial cells before these cells diversified into superkingdoms (Nasir and Caetano-Anollés, 2015, see Schulz et al., 2017 for an opposite view). In an alternative second scenario, the *ABEV* group points to recent HGTs occurring between viruses and cells in either direction, more likely from cell-to-virus considering the cell-like nature of *ABE* FSFs. It is important to note that a single HGT event is sufficient to invoke transformation from the *ABE* to the *ABEV* group. For example, *ABE* FSFs can be transferred directly from Archaea to *archaeoviruses*, from Bacteria to *bacterioviruses*, and Eukarya to *eukaryoviruses*, in addition to indirect cross-superkingdom genetic transfers. All of these transfers suffice for *ABE* to *ABEV* transformation.

**Figure 6:**
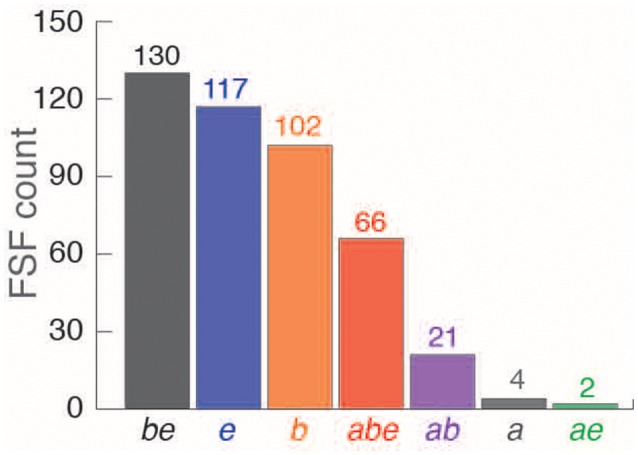
Breakdown of pooled non-redundant 442 viral *ABE* FSFs (see text) into seven possible Venn groups for *archaeoviruses, bacterioviruses*, and *eukaryoviruses*. Numbers on bars indicate actual count (see also Table S6).

To evaluate these alternatives, we pooled the *ABE* FSFs of *archaeoviruses* (*n* = 93), *bacterioviruses* (319), and *eukaryoviruses* (315) into a non-redundant list of 442 FSFs (Table S6). Next, we dissected the 442 FSFs into seven new Venn groups: *a* (*ABE* FSFs detected only in the proteomes of *archaeoviruses*), *b* (*ABE* FSFs detected only in *bacterioviruses*), *e* (*ABE* FSFs detected only in *eukaryoviruses*), *ab* (*ABE* FSFs detected only in prokaryotic viruses), *ae* (*ABE* FSFs detected only in *archaeoviruses* and *eukaryoviruses*), *be* (*ABE* FSFs detected only in *bacterioviruses* and *eukaryoviruses*), and *abe* (*ABE* FSFs detected in *archaeoviruses, bacterioviruses*, and *eukaryoviruses*) (Figure 6). This classification enabled evaluation of virus-to-virus HGTs in contrast to either virus-to-cell or cell-to-virus candidate HGT events postulated above. The majority of the *ABE* FSFs were part of the *be* group (*n* = 130) once again suggesting relatively high activity of cross-superkingdom genetic exchange between *bacterioviruses* and *eukaryoviruses* (or their cellular proteomes) possibly driven by bacteria-eukarya lifestyle affiliations or, as an alternative, loss of these FSFs in *archaeoviruses*. The next larger groups included *e* (117) and *b* (102) (Figure 6). These could represent direct HGT events from bacterial proteomes to *bacterioviruses* and eukaryal proteomes to *eukaryoviruses*, respectively.

A total of 66 *ABE* FSFs were detected in the proteomes of *archaeoviruses, bacterioviruses*, and *eukaryoviruses* (Venn group *abe*), again enriched in cell-like functions (highlighted in Table S6). The *abe “*universal” or “core” group of FSFs therefore included FSFs detected in the proteomes of all virus groups (*archaeoviruses, bacterioviruses*, and *eukaryoviruses*) and the three superkingdoms (Archaea, Bacteria, and Eukarya). While significant cross-superkingdom indirect genetic exchange cannot be ruled out, the possibility of the same HGT event occurring three times independently and in different ecological habitats should be considered unlikely. The origin of *abe* FSFs is therefore better and more parsimoniously reconciled with an origin of modern viral lineages in ancient cells that existed prior to the diversification of cellular life and experienced high levels of genome reduction (Nasir et al., 2012a; Claverie and Abergel, 2016). One interesting observation was the existence of only 2 FSFs belonging to the *ae* group. An origin of Eukarya from within Archaea has recently been postulated following the reconstruction of the genomes of the candidate archaeal phylum “Lokiarchaeota” and “Asgard” archaea (Zaremba-Niedzwiedzka et al., 2017), which harbor several eukaryote-specific proteins (Guy et al., 2014; Spang et al., 2015, see Nasir et al., 2016; Da Cunha et al., 2017 for opposite views). Under this scenario, one should expect stronger affiliation of *eukaryoviruses* with *archaeoviruses*, which however does not materialize in the FSF data.

Finally, 10 and 55 virus-specific FSFs (*V*) were detected in the proteomes of *bacterioviruses* and *eukaryoviruses*, respectively (none in *archaeoviruses*, Figure 2, Table 2). These protein domains represent crucial viral functions involved in viral pathogenicity and virion synthesis and could therefore become hot targets for designing novel therapeutics against contemporary viruses. Their origin however cannot be attributed to cell-to-virus HGT, as these FSFs are completely absent in cellular proteomes. They could originate either directly in viral lineages during replication inside host cells (refer to the “virocell” concept, Forterre, 2011) or represent ancient proteins relics of an early origin of viruses. Testing either of these two scenarios via data-driven approaches remains an open challenge though insights are starting to emerge (Nasir and Caetano-Anollés, 2015).

**Table 2:** Virus specific FSFs (VSFs).

**SCOP ID**	**SCOP *ccs***	**FSF description**	**Virus classification**	**Member families/order**
158974	b.170.1	WSSV envelope protein-like	*eukaryoviruses*	*Nimaviridae*
88648	b.121.6	Group I dsDNA viruses	*eukaryoviruses*	*Polyomaviridae, Papillomaviridae*
101089	a.8.5	Phosphoprotein XD domain	*eukaryoviruses*	*Mononegavirales*
69070	a.150.1	Anti-sigma factor AsiA	*bacterioviruses*	*Caudovirales*
89433	b.127.1	Baseplate structural protein gp8	*bacterioviruses*	*Caudovirales*, Unclassified
160099	d.346.1	SARS Nsp1-like	*eukaryoviruses*	*Nidovirales*
89428	b.126.1	Adsorption protein p2	*bacterioviruses*	*Tectiviridae*
143076	d.302.1	Coronavirus NSP8-like	*eukaryoviruses*	*Nidovirales*
56502	d.172.1	gp120 core	*eukaryoviruses*	*Retroviridae*
55671	d.102.1	Regulatory factor Nef	*eukaryoviruses*	*Retroviridae*
56983	f.10.1	Viral glycoprotein, central and dimerisation domains	*eukaryoviruses*	*Flaviviridae, Togaviridae*
50012	b.31.1	EV matrix protein	*eukaryoviruses*	*Mononegavirales*
118208	e.58.1	Viral ssDNA binding protein	*eukaryoviruses*	*Herpesvirales*
54957	d.58.8	Viral DNA-binding domain	*eukaryoviruses*	*Papillomaviridae, Herpesvirales*
48493	a.120.1	gene 59 helicase assembly protein	*bacterioviruses*	*Caudovirales*, Unclassified
101816	b.140.1	Replicase NSP9	*eukaryoviruses*	*Nidovirales*
48145	a.95.1	Influenza virus matrix protein M1	*eukaryoviruses*	*Orthomyxoviridae*
140506	a.30.8	FHV B2 protein-like	*eukaryoviruses*	*Nodaviridae*
161240	g.92.1	T-antigen specific domain-like	*eukaryoviruses*	*Polyomaviridae*
69922	f.12.1	Head and neck region of the ectodomain of NDV fusion glycoprotein	*eukaryoviruses*	*Mononegavirales*
101156	a.30.3	Nonstructural protein ns2, Nep, M1-binding domain	*eukaryoviruses*	*Orthomyxoviridae*
143021	d.299.1	Ns1 effector domain-like	*eukaryoviruses*	*Orthomyxoviridae*
49818	b.19.1	Viral protein domain	*eukaryoviruses*	*Nidovirales, Orthomyxoviridae, Reoviridae*
75347	d.13.2	Rotavirus NSP2 fragment, C-terminal domain	*eukaryoviruses*	*Reoviridae*
48345	a.115.1	A virus capsid protein alpha-helical domain	*eukaryoviruses*	*Reoviridae*
141666	b.164.1	'SARS ORF9b-like	*eukaryoviruses*	*Nidovirales*
82046	b.116.1	Viral chemokine binding protein m3	*eukaryoviruses*	*Herpesvirales*
56558	d.182.1	Baseplate structural protein gp11	*bacterioviruses*	*Caudovirales*
103145	d.255.1	Tombusvirus P19 core protein, VP19	*eukaryoviruses*	*Tombusviridae*
160892	d.378.1	Phosphoprotein oligomerization domain-like	*eukaryoviruses*	*Mononegavirales*
103068	d.254.1	Nucleocapsid protein dimerization domain	*eukaryoviruses*	*Nidovirales*
51289	b.85.5	Tlp20, baculovirus telokin-like protein	*eukaryoviruses*	*Baculoviridae*
75574	d.216.1	Rotavirus NSP2 fragment, N-terminal domain	*eukaryoviruses*	*Reoviridae*
49894	b.28.1	Baculovirus p35 protein	*eukaryoviruses*	*Baculoviridae, Poxviridae*,
161003	e.75.1	flu NP-like	*eukaryoviruses*	*Orthomyxoviridae*
110304	b.148.1	Coronavirus RNA-binding domain	*eukaryoviruses*	*Nidovirales*
48045	a.84.1	Scaffolding protein gpD of bacteriophage procapsid	*bacterioviruses*	*Microviridae*
58030	h.1.13	Rotavirus nonstructural proteins	*eukaryoviruses*	*Reoviridae*
69652	d.199.1	DNA-binding C-terminal domain of the transcription factor MotA	*bacterioviruses*	*Caudovirales*
58034	h.1.14	Multimerization domain of the phosphoprotein from sendai virus	*eukaryoviruses*	*Mononegavirales*
55064	d.58.27	Translational regulator protein regA	*bacterioviruses*	*Caudovirales*, Unclassified
50176	b.37.1	N-terminal domains of the minor coat protein g3p	*bacterioviruses*	*Inoviridae*
118173	d.293.1	Phosphoprotein M1, C-terminal domain	*eukaryoviruses*	*Mononegavirales*
47724	a.54.1	Domain of early E2A DNA-binding protein, ADDBP	*eukaryoviruses*	*Adenoviridae*
57917	g.51.1	Zn-binding domains of ADDBP	*eukaryoviruses*	*Adenoviridae*
143587	d.318.1	SARS receptor-binding domain-like	*eukaryoviruses*	*Nidovirales*
75404	d.213.1	VSV matrix protein	*eukaryoviruses*	*Mononegavirales*
160957	e.69.1	Poly(A) polymerase catalytic subunit-like	*eukaryoviruses*	*Poxviridae*
140367	a.8.9	Coronavirus NSP7-like	*eukaryoviruses*	*Nidovirales*
160453	d.361.1	PB2 C-terminal domain-like	*eukaryoviruses*	*Orthomyxoviridae*
56548	d.180.1	Conserved core of transcriptional regulatory protein vp16	*eukaryoviruses*	*Herpesvirales*
49889	b.27.1	Soluble secreted chemokine inhibitor, VCCI	*eukaryoviruses*	*Poxviridae*
144251	g.87.1	Viral leader polypeptide zinc finger	*eukaryoviruses*	*Picornavirales*
89043	a.178.1	Soluble domain of poliovirus core protein 3a	*eukaryoviruses*	*Picornavirales, Theilovirus*
144246	g.86.1	Coronavirus NSP10-like	*eukaryoviruses*	*Nidovirales*
47852	a.62.1	Hepatitis B viral capsid (hbcag)	*eukaryoviruses*	*Hepadnaviridae*
69903	e.34.1	NSP3 homodimer	*eukaryoviruses*	*Reoviridae*
159936	d.15.14	NSP3A-like	*eukaryoviruses*	*Nidovirales*
69908	e.35.1	Membrane penetration protein mu1	*eukaryoviruses*	*Reoviridae*
101257	a.190.1	Flavivirus capsid protein C	*eukaryoviruses*	*Flaviviridae*
111379	f.47.1	VP4 membrane interaction domain	*eukaryoviruses*	*Reoviridae*
90246	h.1.24	Head morphogenesis protein gp7	*bacterioviruses*	*Caudovirales*
57647	g.34.1	HIV-1 VPU cytoplasmic domain	*eukaryoviruses*	*Retroviridae*
117066	b.1.24	Accessory protein X4 (ORF8, ORF7a)	*eukaryoviruses*	*Nidovirales*
51332	b.91.1	E2 regulatory, transactivation domain	*eukaryoviruses*	*Papillomaviridae*

*FSFs are identified both by SCOP numeric IDs and alpha-numeric concise classification strings (ccs). See Nasir and Caetano-Anollés (2015) for an expanded list*.

## Discussion

A simple comparative genomic analysis calculating the spread of viral protein domain structure FSFs in reported host and non-host cellular proteomes revealed that proteomes of virus hosts harbored several viral hallmark proteins necessary for virion assembly and successful viral infection cycles (Table 1). These viral hallmark proteins however were absent from the majority of closely-related organisms within the same superkingdom indicating that their rare presence in some host cellular proteomes could be an outcome of virus-to-cell gene transfer. In turn, proteomes not presumed to serve as natural hosts for viruses also shared homologous FSFs with viral proteomes. These FSFs included both viral- and cell-like proteins. This was especially obvious for FSFs shared between *bacterioviruses* and eukaryotic proteomes indicating either direct or indirect cross-superkingdom genetic exchange. This sharing could have been driven by the endosymbiotic and pathogenic lifestyle of bacteria that sometimes associate with eukaryotic cells. Interestingly, despite sharing the same ecosystem with Bacteria (e.g., the human gastrointestinal tract, Lurie-Weinberger and Gophna, 2015), our results suggested that little or no FSF sharing (or genetic exchange) occurred between *archaeoviruses* and the proteomes of Bacteria and Eukarya (e.g., *AB* was 1 and *AE* 0 in *archaeoviruses*, Figure 2). Bacteria are established pathogens and (endo)-symbionts of eukaryotes but Archaea are not known to infect eukaryotic organisms (Aminov, 2013). The membranes of Archaea also differ in lipid composition with the membranes of Bacteria and Eukarya (ether-linked vs. ester-linked, Jain et al., 2014), along with other differences (Gill and Brinkman, 2011). These differences could therefore pose a barrier for *archaeoviruses* to cross/traverse bacterial and eukaryal membranes and participate in horizontal genetic exchange. In contrast and thanks to the relatively similar lipid organization of Bacteria and Eukarya, *bacterioviruses* may either directly traverse eukaryotic membranes or alternatively transduce benign bacterial species into human pathogens by transferring virulence factors (Brüssow et al., 2004), which in turn infect Eukarya. As stated by Gill and Brinkman (2011), “*eukaryotic viruses infect eukaryotes, and bacteriophages transduce Bacteria, which allows them to infect Eukarya”*. Moreover, there are many more known examples of obligate and facultative intracellular bacteria (e.g., *Chlamydia, Rickettsia, Mycoplasma*) in eukaryotes. Therefore, viral infection of bacterial endosymbionts or prophage integration into their genomes will create more opportunities for genetic interactions with *eukaryoviruses* and eukaryotic proteomes explaining more “crosstalk” between *bacterioviruses* and Eukarya (and perhaps *eukaryoviruses* and Bacteria) than between *archaeoviruses* and Bacteria/Eukarya. However, it must be noted that both *archaeoviruses* and archaeal species are relatively underrepresented in sequence databases. Thus, a global picture of the true contribution of *archaeoviruses* and archaeal proteomes to protein structure space remains elusive despite increased metagenome sequencing efforts. Indeed, Archaea constitute an important part of the animal microbiota (Hoffmann et al., 2013; Lurie-Weinberger and Gophna, 2015), an ecosystem that is considered a “hot spot” for genetic exchange (Shterzer and Mizrahi, 2015).

A large cohort of universal protein domains shared between *archaeoviruses, bacterioviruses, eukaryoviruses*, Archaea, Bacteria, and Eukarya, was also detected that provides support to an ancient co-existence of viral and cellular ancestors before the rise of a diversified cellular world (*abe* FSFs, highlighted in Table S6), a scenario supported by a recent large-scale phylogenomic study (Nasir and Caetano-Anollés, 2015) and a number of philosophical arguments (Claverie and Abergel, 2016). Cross-superkingdom genetic transfers were also likely after the rise of diversified cellular lineages. In turn, the list of virus-specific protein domains (Table 2) provides a useful set of molecular targets for antiviral research.

The comparative genomic approach presented here is useful to postulate data-driven hypotheses regarding viral evolution, especially because large-scale sequence-based phylogenetic analysis on viral genes and genomes is sometimes prohibitive due to high nucleotide and amino acid sequence variability within and between viral genome groups. The comparative genomic approach however does suffer from some limitations. First, in the absence of phylogenetic reconstruction, structural similarities are considered homologies. Protein domain sharing could be a result of convergent evolution, HGT and vertical evolution. However, protein domains grouped into FSFs are believed to have evolved from a common evolutionary ancestor and thus cannot (by SCOP definitions) be subject to convergent evolution. Specifically, the interlocking of amino acid side-chains in the buried cores of protein domain structures represents a distinctive “fingerprint,” which is recognizable among member domains of any particular superfamily. Amino acid substitutions that occur over evolutionary timespans do not distort the 3D fingerprint characteristic of each superfamily without risking loss of the protein fold, and ultimately its biochemical function (e.g., bacterial MreB and FtsZ proteins that are prokaryotic homologs of eukaryotic actin and tubulin, respectively). That is the reason why despite low sequence identities, member protein domains of SCOP FSFs share recognizable structural and biochemical similarities, which are taken as evidence for common origin. Empirically, the odds of originating the same fingerprint (a product of multiple interactions occurring between many amino acid side chains) independently are considered to be extremely low (e.g., between 3 and 5% in Gough, 2005). In other words, each known fold or FSF is a unique discovery in evolution. Given the small number of expected folds that exist in nature (~1,500), convergence becomes an unlikely scenario.

Second, HGT can transfer protein domains and thus increase their representation in modern proteomes. We used the *f*-value as a proxy to evaluate the relative evolutionary spread of each FSF in cellular proteomes. When linked with the biochemical function of the protein fold (i.e., viral-like or cellular-like), the analysis indicated a likely direction of gene transfer (i.e., virus-to-cell or cell-to-virus). For example, FSFs involved in a viral hallmark function such as virion synthesis and/or capsid assembly that had negligible presence in either host or non-host superkingdoms (e.g., *f* < 1%) were treated as candidate virus-to-cell gene transfers. In turn, FSFs involved in cellular functions such as metabolism that were widespread among cellular proteomes (e.g., *f* > 60%) were treated as cell-to-virus candidate HGTs, except when these FSFs were also detected in the three virus groups (i.e., the *abe* group of 66 “universal” FSFs). This approach of inferring a qualitative likelihood of HGT is thus similar to the method of detecting anomalous phylogenetic distributions of genes where rare presence of a gene in closely related members is more likely a result of HGT rather than vertical evolution, especially because the latter would require invoking multiple events of gene loss that are less parsimonious than considering fewer HGT events (Philippe and Douady, 2003). The *f*-value approach is especially useful for viral genes that exhibit fast mutation rates and prohibit utilizing genome-scale alignment-dependent phylogenetic analysis (Abroi and Gough, 2011; Nasir and Caetano-Anollés, 2015).

Third, it can be argued that the *f*-value may not reflect phylogenetic diversity. For example, an *f*-value of 0.05 indicates rare presence but the FSF could be specific to a particular phylum or group of organisms (e.g., Firmicutes). However, and to emphasize, the *f*-value was coupled with known biochemical functions of the protein fold, i.e., viral-like (e.g., virion assembly) or cell-like (e.g., metabolism) functions, which was then used as composite variable to postulate the direction of candidate HGT event (see Nasir and Caetano-Anollés, 2013 for previous applications of the approach). When the molecular function of an FSF is well-known (i.e., cell-like or virus-like), it becomes easier to postulate a direction of gene transfer and to also exclude convergence as an alternative scenario. Moreover, FSFs that are specific to only one phylum (or a group of organisms) are likely not to be inherited vertically but after the divergence from the common ancestor of that group, a time period that follows virus-cell divergence.

Fourth, we raise the issue of coverage of viral proteomes, where coverage is defined by the number of viral genes (proteins) with significant homologs in either sequence or structure databases. We have previously shown that roughly >60% of viral proteins did not match to known FSFs (Figure 2B in Nasir and Caetano-Anollés, 2015). It is already well-known that the majority of viral genes lack sequence homologies, putatively termed ORFans (Ogata and Claverie, 2007; Yin and Fischer, 2008; Cortez et al., 2009). These viral genes either evolved fast and hence are no longer recognizable at either sequence or structure levels, or represent genes that originated directly in viruses (Forterre, 2011) (e.g., VSFs in Table 2). Determining the origin of viral ORFans remains an open and important question in virology research.

Finally, we only considered *f*-values of virus-encoded FSFs in cellular proteomes and not in viral proteomes. Viruses are notorious for encoding small-sized genomes that are likely a result of extreme genome reduction (Nasir et al., 2012a; Claverie and Abergel, 2013). In a recent analysis, we showed that only three viral FSFs had an *f*-value of over 0.3 (Nasir and Caetano-Anollés, 2015). This result is unsurprising considering that the 3,440 viruses of this study belong to seven different replication strategies, infect the many diverse groups of cellular organisms (see Nasir et al., 2014 for a mapping of virus replicons to their hosts), and in general harbor genomes and particle sizes that are minimalistic. The tendency of viruses to reduce genome size over long evolutionary timespans has effectively led to loss of information when extant virus genomes are comparatively analyzed with cellular genomes. Indeed, no single FSF could be detected in all seven viral replicon types (Nasir and Caetano-Anollés, 2015). Therefore, we caution the readers that the strategy reported in this study takes a modern-day snapshot of the proteomes of both viruses and cellular organisms and does not benefit from phylogenomic reconstruction. It is also dependent on the size of available genomic databases that are severely under-represented, especially, in archaeal and viral genome sequences. However, we do not expect that sequencing and discovery of novel viral and cellular lineages will drastically compromise our conclusions since we used a very strict threshold (e.g., *f* < 1%) in classifying an FSF to be acquired horizontally from viruses along with investigation of its biochemical function (e.g., virion synthesis). That is, future discovery of a virus-hallmark FSF in hundreds of newly sequenced genomes of a superkingdom that would significantly increase the *f*-values should be considered a highly unlikely event. However, discovery of novel viruses/cellular lineages can definitely add more virus-derived genes in cellular organisms thus adding to the lists of virus-acquired genes in cells or virus-specific genes (Table 2). Moreover, we restricted our analysis to the reference genomes of viruses and corresponding host organisms and to coding DNA. The next logical step is to perform a similar exercise on viruses recovered from metagenomic samples that are increasingly populating bioinformatics databases due to the continuous decline in sequencing cost and availability of fast and reliable high-throughput sequencing platforms. However, it can be sometimes challenging to establish host tropism in metagenome samples. Furthermore, there is no single universal gene (i.e., ribosomal RNA gene in cellular organisms) that can taxonomically classify short sequencing reads of viral metagenomes (Rohwer and Edwards, 2002). That is why we restricted our analysis to only well-curated reference genomes with virus host information available from experimental studies. Similarly, many viral genetic elements are permanently integrated into cellular genomes (Katzourakis and Gifford, 2010). This DNA also originated in viruses and thus should be considered the horizontal transfer of non-coding virus-to-cell transfer. While the virology community remains divided whether or not to include viruses in the realm of life (Claverie and Ogata, 2009; Moreira and Lopez-Garcia, 2009; Forterre, 2016), there have been recent important phylogenomic data-driven breakthroughs unfolding viral origins (Nasir and Caetano-Anollés, 2015). Past events such as FSFs lost via reductive evolution or species extinction leading to loss of ancestral FSFs cannot be accounted for in this analysis without phylogenomic reconstruction. Moreover, inferences drawn in this study are best parsimonious explanations consistent with reported data and lead to testable hypotheses. It is expected that a global dissection of viral and host proteomes will inspire debates and improve studies to better understand viral-host co-evolution.

## Methods

### Data retrieval and manipulation

Proteome data used in this study was taken from Nasir and Caetano-Anollés (2015). In brief, a total of 190,610 protein sequences corresponding to 3,966 completely-sequenced reference genomes of viruses that were available on the NCBI Viral Genomes Resource in June 2014 (Brister et al., 2015) were downloaded. Reference virus genomes are well-curated first genomes submitted for any virus species. Subsequent submissions of new genomes for that virus species are termed “genome neighbors.” In our study, we only kept reference viral genomes, corresponding to any of the seven known viral replicon types (i.e., dsDNA, ssDNA, plus-ssRNA, minus-ssRNA, dsRNA, and retrotranscribing viruses) and excluding genomic neighbors, viruses that were listed as either “unclassified” or “unassigned” and deltaviruses.

### Protein structure assignment

Viral proteins were scanned against a library of hidden Markov models (HMMs) (Gough et al., 2001; Gough and Chothia, 2002) maintained by the SUPERFAMILY database (ver. 1.75) (de Lima Morais et al., 2011) to detect SCOP FSFs using stringent *E*-value cutoff of 10^−4^. Viral genomes with no hits were discarded from the analysis. This reduced the viral dataset to include a total of 3,460 viruses including 1,649 dsDNA, 534 ssDNA, 166 dsRNA, 991 ssRNA, and 120 retrotranscribing viruses. Virus host information was available for 3,440/3,460 viruses (Bao et al., 2004) and was used to identify 62 *archaeoviruses*, 1,223 *bacterioviruses*, and 2,155 *eukaryoviruses* (Table S1). In parallel, pre-calculated FSF assignments for ~11 million proteins encoded by the completely-sequenced genomes of a total of 1,620 cellular organisms including 122 Archaea, 1,115 Bacteria, and 383 Eukarya were retrieved directly from the local installation of SUPERFAMILY MySQL database (July 2014). HMM assignments for viral proteomes can be downloaded from https://figshare.com/articles/Nasir_and_Caetano-Anolles_2015_zip/4833641.

### Calculation of FSF spread in proteomes

The spread of each viral FSF in proteomes was calculated by an *f*-value, which represents the number of proteomes in a superkingdom (or virus group) encoding an FSF divided by the total number of proteomes in that superkingdom (or group). The resulting statistic is given on a scale from 0 to 1 indicating range from either complete absence (i.e., *f*-value = 0) to ubiquitous presence (*f*-value = 1). The index does not evaluate how heterogeneous is that distribution.

### Determination of virus-to-cell and cell-to-virus HGT events

Two factors were considered when postulating the direction of gene transfer (Figure 1): (i) the reported biochemical function of an FSF (e.g., virion synthesis or ATP synthesis), and (ii) spread of that FSF in the proteomes of cellular superkingdom(s). For example, if an FSF involved in capsid assembly (a virus hallmark function) was detected in only few cellular proteomes (e.g., <1%), then this FSF was determined to have transferred horizontally from virus-to-cell. In turn, if an FSF involved in cellular hallmark function (e.g., metabolism) and widespread in cellular proteomes (e.g., >60% presence) was detected in some viral proteomes then this was FSF was determined to have transferred horizontally from cell to viruses. The exception would be the presence of “cell-like” FSFs in the proteomes of all three virus groups, i.e., *archaeoviruses, bacterioviruses*, and *eukaryoviruses*, suggesting a cellular co-existence between viral and cellular ancestors prior to diversification of modern life (Nasir et al., 2012a,b). Thus, both the FSF spread and its biochemical function were considered when postulating the direction of gene transfer.

## Author contributions

AN conceived the study. AN, KMK, and GCA designed the experiments. SSM and SAZ performed the preliminary experiments and wrote the first draft. AN, KMK, and GCA edited and improved the manuscript. All authors approved the study, final manuscript, and conclusions.

### Conflict of interest statement

The authors declare that the research was conducted in the absence of any commercial or financial relationships that could be construed as a potential conflict of interest.

## Supplementary material

Table S1 List of viruses analyzed in this study. Also listed are virus replicon type, virus host, and known family/order.

Table S2 FSFs detected in *archaeoviruses*. FSFs are identified both by SCOP numeric IDs and alpha-numeric *concise classification strings* (*ccs*). The *f*-values are also listed for Archaea, Bacteria, and Eukarya. FSFs shared exclusively with non-host superkingdoms are highlighted.

Table S3 FSFs detected in *bacterioviruses*. FSFs are identified both by SCOP numeric IDs and alpha-numeric *concise classification strings* (*ccs*). The *f*-values are also listed for Archaea, Bacteria, and Eukarya. FSFs shared exclusively with non-host superkingdoms are highlighted.

Table S4 FSFs detected in *eukaryoviruses*. FSFs are identified both by SCOP numeric IDs and alpha-numeric *concise classification strings* (*ccs*). The *f*-values are also listed for Archaea, Bacteria, and Eukarya. FSFs shared exclusively with non-host superkingdoms are highlighted.

Table S5 The composition of *BE* Venn group in *bacterioviruses* and *eukaryoviruses*. FSFs are identified both by SCOP numeric IDs and alpha-numeric *concise classification strings* (*ccs*). The *f*-values are listed for Bacteria, and Eukarya. The difference in *f*-values was calculated by subtracting *f*-value of an FSF in Bacteria from the corresponding *f*-value for that FSF in Eukarya. FSFs with an *f-*value difference of over 25% are highlighted (yellow when *f*-value in Eukarya exceeds that of in Bacteria and blue for *vice versa*). Data sorted in an increasing manner for FSFs common to both *bacterioviruses* and *eukaryoviruses*, unique to *bacterioviruses*, and unique to *eukaryoviruses*.

Table S6 A non-redundant list of 442 *ABE* viral FSFs detected in all three superkingdoms. FSFs are identified both by SCOP numeric IDs and alpha-numeric *concise classification strings* (*ccs*). The *f*-values are also listed for Archaea, Bacteria, and Eukarya, and *archaeoviruses, bacterioviruses*, and *eukaryoviruses*. *abe* FSFs are highlighted.
